# Involvement of MicroRNA in Microglia-Mediated Immune Response

**DOI:** 10.1155/2013/186872

**Published:** 2013-05-23

**Authors:** J. Guedes, A. L. C. Cardoso, M. C. Pedroso de Lima

**Affiliations:** ^1^PhD Programme in Experimental Biology and Biomedicine (PDBEB), CNC - Center for Neuroscience and Cell Biology, University of Coimbra, 3004-517 Coimbra, Portugal; ^2^Institute for Interdisciplinary Research (IIIUC), University of Coimbra, 3030-789 Coimbra, Portugal; ^3^CNC - Center for Neuroscience and Cell Biology, University of Coimbra, 3004-517 Coimbra, Portugal; ^4^Department of Life Sciences, Faculty of Science and Technology, University of Coimbra, 3001-401 Coimbra, Portugal

## Abstract

MicroRNAs (miRNAs) are an abundant class of small noncoding RNA molecules that play an important role in the regulation of gene expression at the posttranscriptional level. Due to their ability to simultaneously modulate the fate of different genes, these molecules are particularly well suited to act as key regulators during immune cell differentiation and activation, and their dysfunction can contribute to pathological conditions associated with neuroinflammation. Recent studies have addressed the role of miRNAs in the differentiation of progenitor cells into microglia and in the activation process, aiming at clarifying the origin of adult microglia cells and the contribution of the central nervous system (CNS) environment to microglia phenotype, in health and disease. Altered expression of several miRNAs has been associated with Alzheimer's disease, multiple sclerosis, and ischemic injury, hence strongly advocating the use of these small molecules as disease markers and new therapeutic targets. This review summarizes the recent advances in the field of miRNA-mediated regulation of microglia development and activation. We discuss the role of specific miRNAs in the maintenance and switching of microglia activation states and illustrate the potential of this class of nucleic acids both as biomarkers of inflammation and new therapeutic tools for the modulation of microglia behavior in the CNS.

## 1. Introduction

Microglia cells are crucial for the development and maintenance of the central nervous system (CNS). In addition to acting as sensors of environmental changes that precede pathological events, these cells have been shown to support neuronal function by monitoring synaptic activity, controlling synaptogenesis, and promoting neuronal apoptosis during development [1–3]. Although they are considered one of the four major cellular types of the CNS, they do not originate from the same precursor cells as astrocytes, oligodendrocytes, or neurons. Instead, they derive from myeloid progenitor cells and share several markers with peripheral monocytes, macrophages, and dendritic cells, such as CD11b, F4/80, and CD45 [4]. The first resident parenchymal microglia cells are believed to originate from yolk sac immature macrophages in early stages of fetal development. In humans, microglia precursor cells arrive at the brain in two waves during the first and second trimester of gestation, while in rodents this migration occurs shortly before and after birth. 

The sudden increase in CD11b^+^ and F4/80^+^ cells observed in the early postnatal period in rodents was until recently attributed to the recruitment of bone marrow derived cells, suggesting that myeloid precursors could also contribute to the initial pool of microglia cells in the CNS. However, most of the studies supporting these findings used irradiation of the recipient animals to allow bone marrow engraftment of genetically-labeled cells [5], which was later found to strongly influence the observed results [6]. In 2010, Ginhoux and colleagues shed light on the origin of microglia. The authors performed *in vivo* lineage tracing studies using Cre recombinase activity, which was induced into pregnant mice between days 7 and 8 of fetal development, when embryonic hematopoiesis is restricted to the yolk sac. The results from this study clearly demonstrated that postnatal hematopoietic progenitors do not contribute significantly to microglia postnatal numbers and that the cellular expansion observed in this period is mainly dependent on the proliferation of the resident yolk sac-derived microglia population [7]. 

The question remains whether this is also true in the adult brain, especially following a neurological insult or in the case of a neurodegenerative disease, wherein the integrity of the blood-brain barrier (BBB) may be compromised. Several studies have shown that the infiltration of bone marrow-derived cells into the brain is possible under those circumstances and may even play a central role in disease modulation. Nevertheless, the exact nature of the contribution of parenchymal and blood-derived microglia to the neuroimmune response, in the context of neuronal disease, remains to be clarified [8–10].

Following their migration to the neuronal tissue, microglia cells assume a surveying phenotype, usually referred as “resting microglia,” characterized by a small and static cell body, a large number of highly motile ramifications, and low expression of macrophage-related surface markers, such as the major histocompatibility complex II (MHC II) and CD45 [11]. The low levels of these markers distinguish parenchymal “resting” microglia from peripheral macrophages. However, following a neuronal insult, such as ischemia, infection, and trauma or in the presence of inflammatory mediators (IFN-*γ*), microglia cells assume an amoeboid form, losing their ramifications, and overexpressing the above-mentioned markers. This process is referred to as microglia “activation” and is known to induce profound phenotypical changes, making parenchymal microglia to become almost indistinguishable from peripheral macrophages [11]. 

Similarly to what has been described in macrophages, microglia activation can also originate different subsets of cells, depending on the nature of the activating stimulus and surrounding environment. These different activation phenotypes express distinct molecular markers and exert different functions in the neuronal tissue [12]. The definition of the different activation states of macrophages was initially based on the expression of proinflammatory receptors and cytokines (M1 phenotype—classical activation) or on the expression of anti-inflammatory receptors and cytokines (M2 phenotype—alternative activation). Further studies revealed the existence of several intermediate activation states, which led to the conclusion that these two basic phenotypes can overlap and that macrophages are able to assume a broad spectrum of phenotypes that cannot be oversimplified and separated into discrete categories. 

Contrarily to macrophages, the mechanisms responsible for microglia phenotype regulation in the CNS are poorly understood. M1 activation, which is characterized by an increase in the production of IL-1*β*, IL-6, TNF-*α*, and nitric oxide (NO), has been identified following acute brain injury caused by trauma or stroke [13, 14]. However, in the context of neurodegenerative disorders, the distinction between the M1 and M2 phenotypes has proven to be more challenging. While several studies have identified the presence of M2 markers, such as TGF-*β* and IL-10, in the brain of Alzheimer's disease (AD) animal models, as well as an increase in the expression of M2 genes AG1 (arginase 1) and CHI3L1/CHI3L2 (chitinase 3-like 1/2) in AD patients [15], inflammation in the human AD brain has also been associated with upregulation of IL-6, IL-1*β*, and TNF-*α*, all markers of the M1 state, in the vicinity of amyloid deposits [16, 17]. Most authors believe that the M2-like phenotypes are less aggressive to the neuronal tissue, promoting tissue repair and phagocytosis of protein aggregates and cell debris, while the M1-like phenotypes are more prone to induce neuronal toxicity by themselves, due to the high expression of inflammatory mediators and NO. Whether human microglia can switch from an M2 to an M1 phenotype with a detrimental effect to the brain is still not clear. However, several studies have pointed to the possibility of microglia “priming,” a phenomenon associated with age and chronic inflammation, in which exposure to low levels of systemic signaling molecules can exacerbate microglia response to a second local stimulus, such as the presence of A*β* aggregates, potentiating the development of tissue damaging phenotypes [16]. Although some of the molecular intervenients and exogenous stressors underlying microglia activation *in vitro* have been identified [18–20], in more complex environments, such as the diseased brain, there is still a lack of answers concerning the molecular mechanisms responsible for microglia phenotypic changes [21]. This led several scientists to propose a role for certain key transcription factors and microRNAs (miRNAs) in these processes [22]. 

## 2. MiRNA Biogenesis and Activity

MiRNAs are transcribed from intragenic or intergenic regions by RNA polymerase II or RNA polymerase III, originating large stem-loop hairpin structures, designated pri-miRNAs [23]. These structures, which are asymmetrically cleaved by an enzymatic complex containing Drosha, a RNAse III endonuclease, originate hairpin-structured precursors designated pre-miRNA [24, 25]. Alternatively, noncanonical pathways for pre-miRNA biogenesis can occur, such as the production of mirtrons, which consist in pre-miRNAs generated through the direct splicing of introns [26]. 

Pre-miRNAs are exported to the cytoplasm by the complex Exportin-5/RanGTP, which recognizes and binds the characteristic overhangs of pre-miRNAs. These precursors are then incorporated into a processing complex containing another RNAse III endonuclease (Dicer), which is responsible for removing the stem-loop of the precursor, originating a double-stranded miRNA molecule with 3′ overhangs (mature miRNA). This duplex molecule is then incorporated into the RNA-induced silencing complex (RISC) or the microribonucleoprotein complex (miRNP), where the strand with the least thermodynamically stable 5′ end is used to activate the complex and guide it to complementary mRNA targets [27]. During miRNA-mediated posttranscriptional gene regulation, the RISC complex, containing the single stranded miRNA template, binds mainly to the 3′ untranslated region (3′ UTR) of the target mRNA, in one or more locations. The complementarity between the mRNA and the nucleotides 2–8 on the 5′ region of the miRNA (seed region) is responsible for this binding and allows the recognition of multiple mRNA targets by a single miRNA molecule [24]. MiRNA-mediated regulation of gene expression is achieved either by translation repression or degradation of the mRNA target molecule [28] and has been associated with many important biological processes, including inflammation, apoptosis, angiogenesis, development, proliferation, patterning, and differentiation.

## 3. MiRNA Role in Microglia Development

The mechanism of miRNA-mediated posttranscriptional regulation is very well suited to the control of fate-changing cellular events, such as differentiation and activation. Since these processes usually involve changes in several proteins and different signaling pathways, such control can be easily achieved through the expression of a single miRNA or a set of specific miRNAs. In addition, miRNAs can directly target transcription factors, the master regulators of most cellular events. A number of studies have revealed that miRNAs may provide a genetic switch to inactivate a set of target genes through the regulation of a specific transcription factor, while miRNA expression can be regulated by transcription factors that bind upstream of pre-miRNA genes [22, 29]. Therefore, these two classes of regulators can work together to orchestrate complex cellular events, such as monocyte-macrophage differentiation and microglia development from primitive macrophages in the yolk sac or bone marrow precursors. 

Two transcription factors, namely, CEBP*α* and PU.1, have been shown to be critical for monocyte/macrophage differentiation and microglia development, CEBP*α* being considered the master regulator of hematopoietic stem cell differentiation. Several miRNAs are controlled directly by CEBP*α*, including miR-223, which is also regulated by the transcription factor NFI-A. However, while CEBP*α* promotes miR-223 expression, NFI-A inhibits the expression of this miRNA [30], which represses NFI-A through a feedback loop. Therefore, when CEBP*α* levels are high, miR-223 expression is enhanced, and NFI-A levels decrease, promoting granulocyte differentiation, but when NFI-A levels are relatively high and miR-223 levels are low, other pathways are favored, such as monocyte differentiation. 

On the other hand, PU.1 is required to promote the skewing of granulocyte-macrophage progenitors to the monocyte lineage. PU.1 levels, although directly controlled by CEBP*α*, have to be relatively high when compared to CEBP*α* levels, to avoid favoring the granulocyte lineage. MiR-424 is upregulated by PU.1 and, through the targeting of NFI-A, upregulates the expression of the M-CSF receptor, skewing differentiation towards the monocyte/macrophage lineage in the bone marrow [31]. Forrest and coworkers have demonstrated that, in addition to miR-424, miR-222, miR-155, and miR-503 play an important role in monocyte differentiation through combinatorial regulation [31]. These authors have shown that when overexpressed, these miRNAs are able to cause cell-cycle arrest and partial differentiation in THP-1 cells (leukemia model). MiR-222 and miR-155 caused G2 arrest and apoptosis, while miR-424 and miR-503 caused G1 arrest and the downregulation of miR-9, an antidifferentiative miRNA, by targeting a site in its primary transcript.

In another recent study, the oncogenic miR-17-92 cluster (which carries six miRNAs: −17, −18a, −19a, −20a, −19b-1, and −92a) has been directly associated with the process of monocyte to macrophage differentiation, in which PU.1 and Egr2 are also involved [32]. Upon differentiation into macrophages, the transcription factor PU.1 was found to induce the secondary determinant Egr2 which, in turn, directly represses miR-17-92 expression by promoting histone H3 demethylation within the CpG island of the miR-17-92 promoter. Conversely, Egr2 itself is targeted by miR-17-92, indicating the existence of a mutual regulatory relationship between miR-17-92 and Egr2 [32]. Given the similarities between macrophages and microglia, it is reasonable to assume that some of these miRNAs and transcription factors also play an important role in microglia differentiation in the brain, and it would be interesting to ascertain whether the same regulatory loops might exist in yolk sac-derived microglia. 

So far, the only study addressing miRNA contribution to microglia development was performed by Ponomarev and colleagues [33, 34]. These authors showed that miR-124, one of the most abundant miRNAs in the brain, is required to maintain the quiescent state of microglia in the brain. By targeting the transcription factor CEBP*α* and the cyclins CDK4 and CDK6, miR-124 is able to reduce the expression of PU.1 and its downstream target, the M-CSF receptor, restricting cellular proliferation and potentiating the differentiation of primitive macrophages to adult microglia in the brain [33]. While during the first two weeks after birth, microglia isolated from the brain presented low levels of miR-124 and a CD45^high^/MHC class II^high^ phenotype, characteristic of active and proliferating cells, adult microglia presented the opposite phenotype, CD45^low^/MHC class II^low^/miR-124^high^ [33, 34]. The authors hypothesized that the high levels of miR-124 observed in adult microglia are a specific consequence of the CNS environment. This idea was based on their observation that sublethally irradiated mice, transplanted with bone marrow GFP^+^ progenitor cells exhibiting a CD45^high^/MHC class II^high^/miR-124^low^ phenotype, presented GFP^+^ CD11b^+^ positive cells in the brain with a CD45^low^/MHC class II^low^/miR-124^high^ phenotype. To confirm this hypothesis, Ponomarev and colleagues cocultured bone-marrow-derived macrophages with astroglial or neuronal cell lines, and they observed, in both cases, a downregulation of MHC class II and CD45 levels as well as an upregulation of miR-124 expression. Several suggestions were made concerning the mechanisms underlying miR-124 upregulation in microglia, including the direct transfer of miR-124 from neuronal cells to microglia through exosomal shuttle vesicles, direct cell-to-cell contact between these two cell types and the release of anti-inflammatory factors, such as CX3CL1 and TGF-*β*, by neuronal cells [34, 35]. 

## 4. MiRNA Role in Microglia Activation

**Figure 1:**
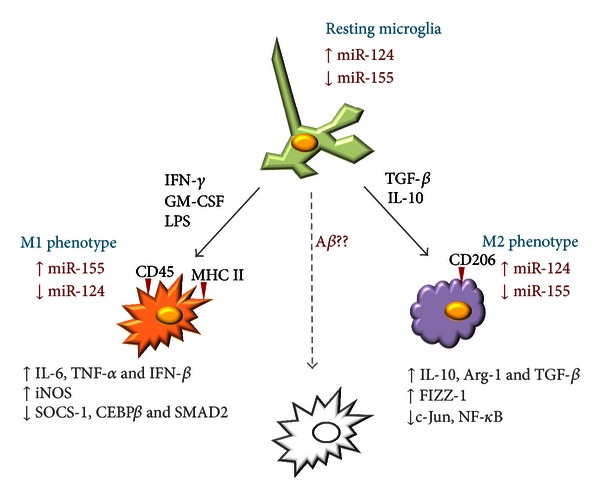
MiRNA contribution to microglia activation phenotypes. Resting microglia cells are characterized by low expression levels of miR-155 and a relatively high expression of miR-124. In the presence of strong inflammatory stimuli, such as IFN-*γ*, GM-CSF, and LPS, microglia assume a classical activation phenotype (M1), characterized by the upregulation of both CD45 and MHC II, the expression of several inflammatory mediators, such as iNOS, the inflammatory cytokines IL-6 and TNF-*α*, the type I interferon IFN-*β*, and the downregulation of miR-124. MiR-155 upregulation is thought to be crucial for the establishment of this phenotype, since this miRNA directly targets several anti-inflammatory molecules, including SOCS-1. Alternatively, in the presence of TGF-*β* or the anti-inflammatory cytokine IL-10, a different activation phenotype is observed (M2). In this case, CD206 is upregulated at the cell surface, and anti-inflammatory molecules involved in tissue repair and angiogenesis are expressed. Moreover, most proinflammatory pathways, including those regulated by the transcription factors c-Jun and NF-*κ*B, are inactivated, and miR-155 upregulation is not observed. Certain endogenous danger signals associated with neurodegenerative disorders, such as A*β* fibrils present in senile plaques of AD patients, can also cause microglia activation, although the exact nature of the observed phenotypic changes is yet to be fully characterized.

In addition to being involved in the regulation of the differentiation process of microglia, several studies suggest a role for miRNAs as modulators of M1 and M2 polarization in both microglia and macrophages. MiR-155, broadly considered a proinflammatory miRNA, was one of the first miRNAs to be directly linked to the M1 phenotype (Figure 1). This miRNA was shown to be upregulated in macrophages, monocytes, and microglia in response to several proinflammatory stimuli, such as LPS, IFN-*γ*, and TNF-*α* [18, 36, 37]. In this regard, we have recently shown that miR-155 targets anti-inflammatory proteins in microglia, such as the suppressor of cytokine signaling 1 (SOCS-1), leading to the upregulation of several inflammatory mediators characteristic of the M1 phenotype, including the inducible nitrogen synthase (iNOS), IL-6, and TNF-*α* [18], as described in Figure 1. In addition, miR-155 upregulation increases the expression of IFN-*β*, which is probably related with a feedback mechanism to control the immune response, since IFN-*β* is known to upregulate the expression of SOCS-1 and IL-10, two important anti-inflammatory mediators [38, 39]. Our results confirm that miR-155 upregulation contributes to a microglia-mediated neurotoxic response, which has been largely associated with the M1 phenotype. Furthermore, recent studies have shown that miR-155 is able to target M2-associated genes, such as that encoding SMAD2, a protein involved in the TGF-*β* pathway [40], and CEBP*β*, a transcription factor important for the expression of IL-10, arginase 1, and CD206 [41], further supporting the hypothesis that miR-155 is required to skew microglia activation to M1-like phenotypes. 

Several other miRNAs have been directly related to the M1 phenotype, including miR-101 and miR-125b. Zhu and coworkers observed an upregulation in miR-101 levels in response to several TLR ligands in macrophages. The overexpression of this miRNA resulted in the downregulation of MAPK phosphatase 1 (MPK-1), promoting the activation of MAPK and the expression of M1-associated proinflammatory cytokines, such as IL-6, TNF-*α*, and IL-1. On the other hand, miR-101 inhibition enhanced MPK-1 expression and decreased p38 and JNK activation [42]. Regarding miR-125b, Chaudhuri and colleagues reported an increase in M1 macrophage activation, with upregulation of MHC class II, CD40, CD80, and CD86, upon overexpression of this miRNA. The authors related these effects to miR-125 targeting of interferon regulatory factor 4 (IRF4) and also observed that, upon forced expression of miR-125b, macrophages adopted an M1 cytotoxic phenotype, presented elevated responsiveness to IFN-*γ*, and were more effective in killing EL4 T-lymphoma tumor cells *in vitro* and *in vivo* [43]. 

In contrast to miR-101 and miR-125b, miR-92a was recently shown to be downregulated in response to the activation of different TLRs, this decrease being necessary to enhance TLR-triggered production of inflammatory cytokines in macrophages [44]. On the other hand, miR-92a overexpression inhibited the activation of the JNK/C-JUN pathway through the targeting of mitogen-activated protein kinase kinase 4 (MKK4), which activates JNK/stress-activated protein kinase and a direct target of miR-92a. Therefore, miR-92a downregulation can be considered as an additional requirement for M1 macrophage activation [44].

Finally, as illustrated in Figure 1, miR-124 has been reported to contribute to the M2 phenotype of macrophages and microglia, since its forced overexpression led to the downregulation of M1-associated markers, such as IL-6, TNF-*α*, and iNOS, and to an increase of proteins associated with the M2 phenotype, as is the case of TGF-*β*, arginase-1, and FIZZ1 [33]. It was concluded that, in microglia, an inverse correlation exists between the expression of M1 activation markers and that of miR-124 (Figure 1). Indeed, the highest levels of miR-124 were detected in CD45^low^/MHC class II^low^ nonactivated resident microglia, but miR-124 expression decreased dramatically upon cell treatment with IFN-*γ* and GM-CSF, two stimulus known to upregulate CD45 and MHC class II expression and to potentiate the M1 phenotype [33].  Table 1 lists several miRNAs involved in microglia activation, indicating the phenotype (M1 or M2) favored by their up- or downregulation. 

Taken together, the above-mentioned results highlight the prominent role of miRNAs in the regulation of the activation of both microglia and macrophages and open new possibilities in the field of anti-inflammatory therapies. Using appropriate gene therapy tools, miRNA modulation could be an interesting and promising strategy to fine-tune the immune response, skewing cell activation to the M1 or M2 phenotypes according to the specific requirements of each disease setting. 

## 5. MiRNA Dysregulation in Neurodegeneration and Neuroinflammation

Neurodegeneration is characterized by neuronal loss of specific neuronal circuits associated with cognitive and motor functions. In this context, neuronal death is considered both cause and consequence of neuroinflammation, a process involving microglia and astrocyte proliferation and activation. The excessive production of inflammatory mediators by these cells propagates inflammation in the brain and contributes to the triggering of a local and systemic immune response, which is characterized by changes in miRNA levels in the nervous tissue and in the periphery. Although the traffic of peripheral mononuclear cells from the blood stream to the CNS is tightly regulated at the level of the BBB, it is known that the integrity of this barrier is highly compromised in severe brain diseases, such as stroke, brain trauma [9], and AD [5, 45, 46]. The local disruption of the BBB in a disease context facilitates the passage of blood-derived cells to the nervous tissue. Therefore, both microglia and peripheral mononuclear phagocytes are able to impact the pathoetiology of neurodegenerative diseases, and miRNAs presenting altered expression levels in both of these cell types can be used as new potential biomarkers and targets of neurodegeneration. 

Through the cellular crosstalk between the brain and the periphery, the nervous system can influence peripheral immune functions, and, conversely, the immune system can affect neuronal activity [47]. A specific subset of miRNAs is able to regulate both cognitive and immune features, but the neuroimmune impact of these molecules is far from being fully understood. However, the role of miRNAs in neuroimmune pathologies has recently started to be unveiled. In the past few years, a large number of studies have emerged reporting the disruption of miRNA expression during neuroinflammation and neurodegeneration processes [48]. For example, miR-146, which has been directly related with AD [49] and epilepsy [50], has also been shown to be highly overexpressed in A*β* and TNF-*α* stressed human microglia cells, and this effect was inversely correlated with the levels of inflammation-related proteins, such as CFH and IRAK-1 [51]. Another brain enriched miRNA, miR-124, which has been directly related with the maintenance of the quiescent state of microglia [33], has also been shown to inhibit the neuronal transcription regulator complex REST, which is involved in Rett syndrome [52] and neuronal changes during chronic cocaine intake [53]. 

MiRNA dysregulation in the context of neurodegeneration can be a consequence of genetic or sporadic anomalies. In addition, the miRNA-related machinery can be impaired in neurological disorders [54]. Accordingly, altered miRNA profiles have been identified in several neurodegenerative diseases. Striatal miR-22, miR-29c, miR-128, miR-132, miR-138, miR-218, miR-222, miR-344, and miR-674-3p, as well as the cellular levels of Drosha were shown to be downregulated in both YAC128 and R6/2 transgenic mouse models of Huntington's disease (HD) [55]. In the frontal cortex and striatum of human HD brains, several miRNAs were found to be altered with respect to the brains of healthy subjects, and changes in the primary nucleotide structure of the 3′ terminus of certain miRNAs were also reported [56]. MiRNA profiling studies in Parkinson's disease (PD) revealed an early downregulation of miR-34b/c in human brain areas, with variable neuropathological effects at clinical (motor) stages [57], while in ALS mouse models deficiency in miR-206 accelerated disease progression [58]. 

A large number of studies using cellular and mouse models, human hippocampus, human cortex, and whole brain samples have revealed altered miRNA expression profiles in AD. MiR-106b was found to be aberrantly expressed in the APPswe/PSΔE9 AD mouse model, and its levels were correlated with the dysregulated expression of the TGF-*β* type II receptor [59]. Wang and coworkers have shown that the exposure of SH-SY5Y cells to A*β*_1-42_ oligomers leads to the increase of miR-106b expression and to the consequent impairment in TGF-*β* signaling, providing a possible explanation for the observed neurodegeneration [59]. As mentioned before, TGF-*β* signaling is a marker of the M2 microglia phenotype, and, as such, miR-106b overexpression could be considered an additional cause of microglia skewing to the M1 phenotype following A*β* exposure. 

Interestingly, Schonrock and collaborators have shown that half of the tested miRNAs in their study were downregulated in hippocampal neuronal cultures in response to A*β*_1–42_ peptides and that the dysregulated miRNAs were likely to affect target genes belonging to signaling pathways known to be disrupted in AD. These results were further validated in the hippocampus of APP23 mice and human AD brains [60]. Curiously, many of those miRNAs, as miR-21, miR-146a, let-7i, and miR-125b, had already been associated with inflammation. 

Although the majority of miRNA profiling studies in neurodegenerative diseases has been performed in brain samples, miRNA dysregulation has been found in other biological sources, such as plasma, peripheral blood, and cerebral spinal fluid (CSF). For example, miR-34a was shown to be significantly increased in plasma from HD gene carriers prior to symptom onset, suggesting that plasma miRNAs can be used as biomarkers in HD [61]. In another study, eighteen miRNAs were found to be differentially expressed in peripheral blood mononuclear cells of nineteen PD patients with respect to miRNA levels in thirteen control subjects [62]. In blood serum from AD patients, miR-137, miR-181c, miR-9, and miR-29a/b were found to be downregulated, and although the ability of these miRNAs to conclusively diagnose AD is currently unknown, these blood-circulating miRNAs have potential to be used as additional biomarkers of the disease [63]. Although more difficult to obtain, CSF has also been considered a source of biomarkers for many neurological diseases, since this fluid constantly exchanges material with the brain parenchyma and is less prone to the influence of peripheral organs. Alexandrov and collaborators analyzed miRNA abundance in the CSF of AD patients and observed a significant increase in the levels of miR-9, miR-125b, miR-146a, and miR-155, with respect to age-matched controls. Interestingly, these miRNAs are NF-*κ*B-sensitive proinflammatory miRNAs, also known to be upregulated in AD brains and have been associated with progressive spreading of neuroinflammation [64]. Taken together, these results indicate that the effective application of miRNAs as biomarkers for neurodegenerative diseases should include miRNA profiling, not only in the blood but also in serum, plasma, and different cellular subtypes, as well as the parallel correlation of the obtained results with brain morphology overtime, in order to overcome clinical issues related with disease staging and progression.

MiRNA deregulation has also been associated with viral-induced neuroinflammation and neurodegenerative processes. Mishra and coworkers showed that human microglia cells treated with HIV-1 Tat-C protein, a molecule involved in neuroinflammation in HIV-infected patients, present increased miR-32 expression with consequent changes in the levels of the downstream target TRAF3, which, in turn, was found to control IRF3 and IRF7 expression [65]. It was also demonstrated that miR-146a is upregulated in human microglia cells under HIV infection, regulating the inflammatory response by targeting the CCL8 [66] chemokine. Therefore, it seems that miRNA expression is altered after inflammation in immune and glial cells in order to support the fine-tuning of immune functions essential to maintain brain homeostasis. An interesting study by Dave and Khalili showed that morphine-induced inflammation and oxidative stress in human monocyte-derived macrophages contribute to the expansion of the HIV-1 viral reservoir in the CNS and HIV-associated dementia [67]. The authors provided evidence that this process is regulated by differentially expressed miRNAs, including miR-15b and miR-181b, both linked to several targets in the proinflammatory pathways. 

MiRNAs are also believed to modulate microglia inflammation after hypoxia/ischemia, which may contribute to neuronal damage. Hypoxia causes upregulation of the Fas ligand (FasL) and simultaneously downregulation of miR-21 in microglia, influencing neuronal apoptosis. Interestingly, according to the work of Zhang and colleagues, the ectopic expression of miR-21 partially protects neurons from cell death caused by hypoxia-activated microglia [68]. The same authors reported a potential role for miR-181c in the regulation of TNF-*α* expression after hypoxia/ischemia and microglia-mediated neuronal injury. They showed that oxygen-glucose deprivation (OGD) induces microglia activation *in vitro* and in the hippocampus of Wistar rats (four-vessel occlusion—4-VO—rat model of ischemia), as concluded by observation of the overproduction of TNF-*α*, IL-1*β*, and NO and the downregulation of miR-181c. On the other hand, the heterologous expression of this miRNA was found to protect neurons from cell death caused by OGD-activated microglia [69].

Regarding neurodegenerative pathologies with a known inflammatory component, such as multiple sclerosis (MS), miRNAs have also been shown to play a central role in the regulation of microglia-mediated immune responses. Ponomarev and coworkers have demonstrated that miR-124 is able to switch microglia from an inflammatory to a quiescent state, and this phenomena was considered essential to successfully inhibit the onset of EAE [33]. Also in MS, miR-155^−/−^ knockdown mice were shown to present significantly reduced numbers of encephalogenic CD4^+^ Th17 cells, an inflammatory T-cell subset with an important role in this disease [70]. In a very recent study, Butovsky et al. showed that the modulation of the cytokine profile of inflammatory monocytes using an anti-Ly6C mAb reduced monocyte recruitment to the spinal cord, decreased neuronal loss, and extended survival in a mouse model of ALS. Interestingly, in humans with ALS, the analogous CD14^+^CD16^−^ monocytes were shown to exhibit an ALS-specific miRNA inflammatory signature, which can be used as a biomarker of this disease and reveal novel therapeutic targets [71]. 

Another interesting effect associated with miRNAs derives from their capacity to activate receptors by themselves, after being secreted by cells within the CNS, which allows them to function as signaling molecules. For example, let-7 is known to induce neurodegeneration by binding to TLR7 in neurons and microglia. Let-7 is increased in the CSF of AD patients, and accordingly, the injection of this miRNA in the CSF of wild-type animals caused neurodegeneration, which did not occur in mice lacking TLR7 [72]. Also in the context of AD, the NF-*κ*B-dependent miR-146a was reported to be upregulated in the brain of AD patients and to enhance inflammation by targeting the complement factor H (CFH) [49]. MiR-146a was also found to be overexpressed in prion-infected mouse brain tissues, concurrent with the onset of prion deposition and microglia activation, which suggests that this miRNA plays a role in the proinflammatory response of microglia to prion replication [73]. 

The above-mentioned studies stress the role of miRNAs as modulators of both neuroinflammation and neurodegeneration and illustrate their potential as biomarkers and novel therapeutic targets in CNS diseases. 

## 6. MiRNA-Based Therapeutic Applications in Neuroimmune and Neurodegenerative Diseases

Over the last few years, there has been a significant progress in the development of strategies to modulate the levels of certain miRNAs and miRNA clusters, aiming at adjusting cellular functions dysregulated in several pathologies. Modulation of mature miRNAs can be accomplished by oligonucleotides complementary to miRNA sequences (miRNA inhibitors), causing miRNA knockdown or, alternatively, by miRNA precursor oligonucleotides (miRNA mimics), which cause miRNA overexpression [74–76]. Usually, these oligonucleotides present chemical modifications, such as the incorporation of locked nucleic acids (LNA) nucleotides, which have a methylene bridge between the 2′-oxygen and the 4′-carbon of the ribose moiety or the incorporation of 2′-O-methyl modified nucleotides (antagomirs) in certain positions of the oligonucleotide sequence. The purpose of these modifications is to increase the chemical stability and resistance of the miRNA inhibitors or mimics to serum nucleases, thus potentiating their therapeutic potential. However, in order to achieve a successful clinical application of miRNA-based therapies it is crucial that these oligonucleotides are properly delivered by vehicles that not only reliably and effectively overcome cellular and physiological barriers but are also highly target specific. The presence of the BBB, which restricts entry of therapeutic molecules into the brain, as well as the possible degradation of nucleic acids by nucleases present in the blood, constitutes major obstacles associated with nucleic acid delivery *in vivo*. Nonviral vectors have been developed to ensure protection and improvement of nucleic acid delivery into target cells and have been employed in miRNA-based therapeutic approaches to modulate neuroinflammatory signaling pathways [77]. 

**Figure 2:**
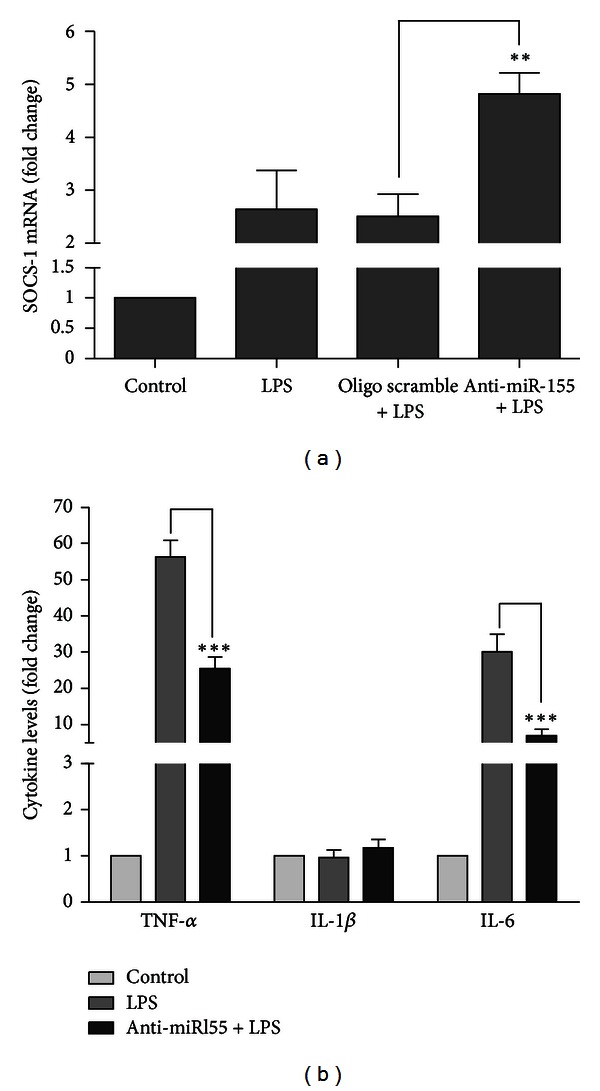
MiR-155 inhibition in microglia cells increases SOCS-1 levels and decreases the release of inflammatory cytokines to the extracellular medium. N9 mouse microglia cells were transfected with anti-miR-155 oligonucleotides (anti-miR155) or with nontargeting oligonucleotides (oligo scramble) complexed with cationic liposomes for 4 h. Twenty-four hours after transfection, cells were incubated with LPS at 0.1 *μ*g/mL for 18 h. The cell culture medium was then collected to determine cytokine protein levels, and total RNA was extracted from the cultured cells. (a) SOCS-1 mRNA levels were quantified by qRT-PCR. (b) The levels of TNF-*α*, IL-1*β*, and IL-6 secreted to the cultured medium were determined by ELISA. Results are expressed as fold change of mRNA or protein levels with respect to control (untransfected and untreated cells). **P < 0.01 compared to cells transfected with the scramble oligonucleotide and ***P < 0.01 compared to LPS-treated cells in the absence of transfection. Results in (a) and (b) are representative of 3 independent experiments performed in triplicate.

In our own work, we have shown that the use of a nonviral strategy to promote miR-155 silencing in microglia, prior to their activation, is able to reduce the release of NO and other inflammatory mediators, such as the major inflammatory cytokines TNF-*α* and IL-6 to the extracellular environment, through an increase in the levels of SOCS-1, a direct target of miR-155 (Figure 2). The modulation of this miRNA in microglia cells, prior to their activation with LPS, proved to be enough to improve cell viability in neuronal cultures incubated with microglia conditioned medium [18]. These results stress the neuroprotective potential of miR-155 silencing in the context of neuroinflammation. 

Other miRNAs have been related with the regulation of the neuroimmune response. As discussed before, miR-124 is one of the main miRNAs responsible for the maintenance of the quiescent state of microglia/macrophages in the brain and spinal cord, and therefore, miR-124 may constitute an important target for the development of therapeutic approaches towards the control of neuroinflammation. Recently, Willemen and coworkers investigated the contribution of miR-124 to the regulation of hyperalgesia and microglia/macrophage activation in LysM-GRK2^+/−^ mice, in which the expression of the G protein-coupled receptor kinase (GRK) 2 is decreased to about 50% in microglia/macrophages, with respect to wild-type animals. These mice develop inflammatory hyperalgesia caused by activation of microglia/macrophages in the spinal cord. The authors showed that the pathological transition from acute to persistent hyperalgesia is associated with reduced levels of miR-124 in spinal cord microglia and with a microglia M1 phenotype switch, leading to increased production of proinflammatory cytokines. The intrathecal administration of miR-124 mimics prevented completely the transition to persistent pain in LysM-GRK2^+/−^ mice and normalized the expression of microglial M1/M2 markers, suggesting that miR-124 administration may be a promising approach to prevent and treat persistent inflammatory and neuropathic pain [78]. 

Another relevant study on miRNA therapeutics in the context of neuroinflammation was reported by Selvamani and coworkers. The authors hypothesized that miRNAs able to target proteins from the insulin-like growth factor-1 (IGF-1) signaling family could be suppressed to promote the neuroprotection provided by IGF-1 following stroke. Using middle-age female rats in which the treatment with estrogen is known to be neurotoxic, the authors administered stereotactically LNA antisense oligonucleotides against miR-1 or let-7f, 4 h after stroke, and observed that miRNA inhibition extended the neuroprotection afforded by IGF-1. Interestingly, although let-7f is a proinflammatory miRNA preferentially expressed in microglia from the ischemic hemisphere, where the IGF-1 expression is detectable, the levels of IGF-1 increased even further in microglia after anti-let-7f treatment [79]. Finally, a study from Lukiw and coworkers showed that the expression of miR-146a is increased in AD brains, which was correlated with a decrease in the CFH expression, a protein responsible for the repression of the inflammatory response in the brain. The inhibition of miR-146, achieved through delivery of a specific LNA-antisense oligonucleotide in human neural cells subjected to oxidative stress, was found to restore CFH expression levels, which were decreased following oxidative damage [49]. 

Taken together, these reports illustrate the vast neuroprotective potential of miRNA-based therapeutic strategies using anti-miRNA oligonucleotides targeting neuroinflammation and confirm the important role of miRNAs in modulating neuroimmune responses to acute and chronic brain damage. Although less explored, the application of miRNA mimics, in order to restore miRNA expression and decrease the levels of potentially neurotoxic proteins, is also an interesting possibility with high therapeutic potential. Nevertheless, it is important to mention that, due to incomplete pairing, each miRNA has the ability to target multiple genes simultaneously, which significantly increases the risks of unspecific effects on miRNA-based therapeutic approaches. To avoid this drawback, it will be relevant to consider not only the strength of the binding between a certain miRNA and all its available targets in a specific tissue but also the relative amounts of these targets and the thermodynamic stability of each miRNA : mRNA duplex [80]. 

## 7. Conclusion

Although the exact nature of the contribution of microglia and peripheral mononuclear phagocytes to neurodegeneration remains to be fully elucidated, several benefits have been suggested for the use of these immune cells in therapeutic strategies designed to curb amyloidosis, decrease EAE progression, fight CNS viral infection, or assist in the reduction of neuroinflammation associated with neurodegenerative diseases. On the other hand, several groups have identified signs of chronic activation and functional impairment in microglia cells isolated from different brain disease models. Given their important role in the regulation of gene expression, we believe that miRNA-based therapies could constitute an interesting and attractive strategy to improve microglia activity, modulating signaling pathways linked with neuroinflammation. In addition, the compromised activity of the BBB in most neurodegenerative disorders, the lower activation threshold of peripheral mononuclear phagocytes compared to brain-derived microglia, and their easy access in a clinical context make these cells another excellent tool for future gene therapy approaches for brain disorders. Overall, understanding the contribution of brain- and blood-derived immune cells to microglia pools in the CNS in a disease context will be of great importance to improve the existing immunotherapies and generate new and effective therapeutic strategies for these diseases.

The fine-tuning activity of miRNAs has been proven crucial in the regulation of differentiation of microglia allowing the maintenance of brain homeostasis. Since a single miRNA has the capacity to target more than one protein involved in the same signaling pathway, their modulation can significantly change cell phenotypes that depend on the levels and activation of specific proteins. Such capacity reflects a molecular paradigm suitable for therapeutic intervention. Due to the lack of minimally invasive diagnosis tools and effective therapeutic options for most CNS diseases, we believe that the use of miRNAs, both as disease biomarkers and therapeutic targets associated with cells of the immune lineage, although yet poorly explored, will tend to grow in the near future. 

## Figures and Tables

**Table 1:** MiRNAs in microglia and macrophage activation.

	Role	Phenotype	Ref
↑ miR-155	Targets SOCS-1 causing ↑ of iNOS, TNF-*α*, IL-6, and IFN-*β* Targets SMAD2 and CEBP*β*	M1	[18, 36, 40]

↑ miR-124	Lead to ↓ IL-6, iNOS, and TNF-*α* and ↑ TGF-*β*, arginase-1, and FIZZ1	M2	[33, 34]

↑ miR-101	↓ MPK-1 promoting MAPK activation and IL-6, TNF-*α*, and IL-1 expression	M1	[42]

↑ miR-125b	↑ MHC class II, CD40, CD80, and CD86 through targeting of IFR4 Elevates responsiveness to IFN-*γ*	M1	[43]

↓ miR-92a	Inhibited activation of JNK/c-Jun through targeting of MKK4 Its downregulation is necessary to promote the M1 phenotype	M1	[44]
